# Causal relationship between obesity and diabetic neuropathy: A 2-sample Mendelian randomization study

**DOI:** 10.1097/MD.0000000000047842

**Published:** 2026-02-28

**Authors:** Zhuolin Wang, Cong Li, Lihui Wang, Xiaoting Zhu, Chengyan Wang, Chunmei Dai, Mingquan Li

**Affiliations:** a Changchun University of Traditional Chinese Medicine, Changchun, China; b The Third Clinical Hospital of Changchun University of Traditional Chinese Medicine, Changchun, China; c The First Clinical Hospital of Jilin Academy of Chinese Medicine Sciences, Changchun, China; d Hospital Management Office, Changchun University of Chinese Medicine, Changchun, Jilin Province, China.

**Keywords:** causal association, diabetic neuropathy, Mendelian randomization, obesity

## Abstract

A growing body of evidence suggests a potential correlation between obesity and diabetic neuropathy (DN), but the causal nature of this relationship remains uncertain. We conducted a Mendelian randomization (MR) analysis to investigate the causal relationship between obesity and DN. Independent single nucleotide polymorphisms strongly associated with DN were identified from genome-wide association studies. Summary statistics for body mass index (BMI), waist circumference (WC), hip circumference (HC), and waist-to-hip ratio (WHR) were also obtained from genome-wide association studies datasets. The causal effects were estimated using inverse-variance weighted analysis, MR-Egger regression, and weighted median estimation. Sensitivity analyzes were performed to assess the robustness of the findings. Inverse-variance weighted analysis revealed significant negative associations of BMI (*P* = .0006, OR = 0.809, 95% CI: 0.716–0.913), WC (*P* = .008, OR = 0.792, 95% CI: 0.665–0.943), and HC (*P* = .032, OR = 0.871, 95% CI: 0.768–0.988) with DN risk. No significant association was observed for WHR (*P* = .425, OR = 0.954, 95% CI: 0.849–1.071). After Bonferroni correction (α = 0.0125), there was a statistically significant relationship between BMI and DN (*P* ≤ .0125), indicating a causal relationship. However, the causal relationships between WC, HC, WHR, and DN were not statistically significant, with *P*-values all > .0125. However, the OR value of MR analysis is close to 1, indicating that BMI may have a slight protective effect on the risk of DN.

## 1. Introduction

Numerous observational studies have reported an association between obesity and diabetic neuropathy (DN).^[1–5]^ This link is of considerable public health importance, given the high and rising prevalence of both obesity and DN worldwide. By 2021, an estimated 529 million individuals were living with diabetes mellitus (DM) globally,^[6]^ and the International Diabetes Federation projects this number will reach 782 million by 2045.^[7]^ Alarmingly, up to 50% of individuals with DM are expected to develop DN.^[8]^ According to the World Atlas of Obesity, by 2030, 34% of the global population will be classified as overweight or obese, including 18% as obese (body mass index [BMI] ≥ 30 kg/m^2^), 6% as severely obese (BMI ≥ 35 kg/m^2^), and 2% as morbidly obese (BMI ≥ 40 kg/m^2^).^[9]^

DN is a common and serious complication of DM, encompassing a spectrum of neurological syndromes. It often develops insidiously, with clinical manifestations resembling those of other neuropathic disorders.^[10]^ Common symptoms include numbness, tingling, pain, and abnormal sensations in the extremities.^[11]^ In advanced cases, foot ulcers and infections may occur, leading to an increased risk of amputation and a marked reduction in quality of life. DN is further associated with high morbidity and mortality, imposing substantial economic burdens on patients and their families.^[12]^ Known risk factors and comorbidities include DM duration and management, arterial hypertension, peripheral arterial occlusive disease, diabetic retinopathy and nephropathy, depression, visceral obesity, hyperlipidemia, and demographic variables such as age, height, and weight.^[13]^

The pathogenesis of DN is complex and multifactorial. Low-grade intraneural inflammation has been identified as a hallmark of DN and may represent a convergent pathway leading to intraepidermal nerve fiber degeneration.^[14]^ Even in the absence of overt diabetes, obesity can induce mild peripheral nerve inflammation, which is linked to neuropathy.^[15]^ Traditional measures of obesity include BMI, waist-to-hip ratio (WHR), waist circumference (WC), and hip circumference (HC). Some studies have demonstrated a correlation between BMI and DN development,^[16]^ but DN-specific research remains limited. Moreover, the causal nature of this relationship remains unclear, as confounding factors cannot be fully excluded in observational designs.

Clarifying the causal relationship between obesity and DN is critical for advancing our understanding of DN pathogenesis and informing effective prevention and treatment strategies. While observational studies have provided valuable insights, their susceptibility to confounding limits causal inference. Mendelian randomization (MR) leverages genetic variants associated with exposure traits as instrumental variables (IVs), exploiting the random allocation of alleles during gamete formation to minimize confounding and reverse causation.^[17]^ In this study, we applied a MR approach to investigate the causal relationships between multiple obesity indicators (BMI, WHR, WC, and HC) and DN. Our findings aim to provide a genetic basis for early prevention and targeted intervention strategies for DN.

## 2. Materials and methods

### 2.1. Research design

This study employed a multiple-exposure MR design. Single nucleotide polymorphisms (SNPs) significantly associated with BMI, WC, HC, and WHR were selected as IVs. DN served as the outcome variable, adopted a 2-sample univariate MR design. All analyzes were performed in the R programming language using the TwoSampleMR package. The robustness of the results was evaluated through Cochran *Q* heterogeneity test, multiple validity checks (MR-Egger regression, MR-PRESSO analysis), and sensitivity analyses (leave-one-out).

**Figure 1.**
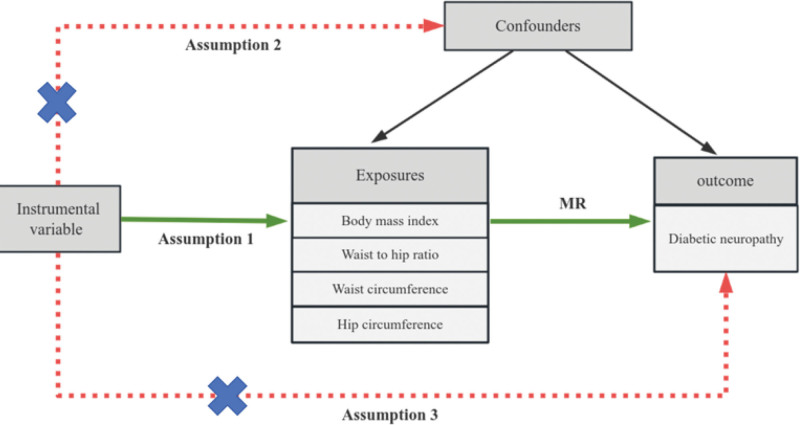
Schematic diagram of MR analysis

The MR approach is based on 3 principal assumptions^[18]^: the IV is strongly associated with the exposure variable. The IV is independent of potential confounders. The IV influences the outcome exclusively through its effect on the exposure (Fig. 1).

### 2.2. Data sources

BMI and WC are among the most commonly used indicators of obesity. According to the World Health Organization, obesity is defined as a BMI ≥ 30 kg/m^2^, while the WHR, calculated as WC (cm)/HC (cm), is considered an indicator of central obesity. Accordingly, we obtained data on BMI (n = 394,642), WHR (n = 502,773), WC (n = 394,642), and HC (n = 394,642) as exposure variables from the genome-wide association studies (GWAS) catalog (https://www.ebi.ac.uk/) and qualified datasets.

Data for the outcome variable (disease phenotype DN) were sourced from the FinnGen database (https://www.finngen.fi/en). The specific phenotypic code is “B-DM_NEUROPATHY,” which is defined based on the International Classification of Diseases (ICD) code. The inclusion criteria are patients with clinically diagnosed DN (including types such as peripheral neuropathy and autonomic neuropathy, not limited to painful neuropathy). The exclusion criteria were non-DN, unclear diagnosis or incomplete clinical data. The sample size of the dataset was 217,735 cases, among which 358 cases were in the case group (confirmed by clinical symptoms, neuroelectrophysiological examination and imaging examination), and 217,377 cases were in the control group (those without a history of diabetes or symptoms and signs related to neuropathy). All datasets were derived from European populations, including both male and female participants. A summary of the data is provided in Table 1.

**Table 1:** Characteristics of GWAS summary data in the study.

Phenotypes	Year of publication	Codes, references or ICD codes	Sample size	Cases (n)	Controls (n)	Cases (%)	Population
Obesity constitution-related phenotypes							
BMI	2025	GCST90468161 (PMID: 39789286)	394,642	NA	NA	NA	European
WHR	2018	GCST90029009 (PMID: 29892013)	502,773	NA	NA	NA	European
WC	2025	GCST90468182 (PMID: 39789286)	394,642	NA	NA	NA	European
HC	2025	GCST90468170 (PMID: 39789286)	394,642	NA	NA	NA	European
Disease							
DN	2021	finn- b-DM_NEUROPATHY	217,735	358	217,377	NA	European

BMI = body mass index, DN = diabetic neuropathy, GWAS = genome-wide association studies, HC = hip circumference, PMID = PubMed ID, WC = circumference, WHR = waist-to-hip ratio.

### 2.3. Statistics of variables

The selection of IVs was strictly based on the correlation with obesity-related traits (BMI, WC, HC, WHR), rather than DN-related SNPs. After retrieving the datasets, we used the R programming language to filter and process the data, thereby minimizing potential analytical bias arising from linkage disequilibrium (LD) imbalances prior to SNP selection. Equivalent SNPs from exposure-related GWAS datasets were selected according to the following criteria: genome-wide significance threshold of *P* < 5 × 10^−8^. Physical distance of at least 10,000 kb between any 2 SNPs. LD threshold of *R*^2^ < 0.01 and an *F*-statistic > 10. SNPs that met these criteria (being independent of each other and strongly associated with the exposure variables) were retained as the final IVs.

### 2.4. Allele coordination program

To ensure that the allele orientations of exposure and outcome effects are consistent and to prevent the reversal of effect directions, the following coordination steps are adopted: unify the reference alleles of SNPs in the exposure and outcome datasets (with GRCh37/hg19 as the reference genome); identify the effectant alleles of each SNP (alleles positively correlated with obesity traits in the exposure dataset and alleles related to the risk of DN in the outcome dataset); for the chain flip problem (such as A/T in the exposed data and T/A in the ending data), correction is made through the flip effect value symbol; eliminate SNPs whose allelic matching relationships cannot be clearly defined; for SNPs missing between the exposure and outcome datasets, if there are highly linked disequilibrium (*R*^2^ > 0.8) alternative SNPs, such alternative SNPs shall be adopted as IVs. If there is no suitable alternative SNP, the deletion variation should be directly excluded to ensure the accuracy of the analysis.

### 2.5. MR analysis

All analyzes were performed in R version 4.4.2, after Bonferroni correction, the test level was α = 0.0125 (due to the involvement of 4 exposure factors, the original α = 0.05, and after correction, α = 0.05/4 = 0.0125). MR was conducted using the TwoSampleMR package, employing 3 main methods: inverse-variance weighted (IVW), MR-Egger regression, and weighted median estimation. The IVW method, the earliest and most widely applied in MR studies, was selected as the primary approach in this study, as it calculates the weighted average of effect sizes across all IVs. MR-Egger regression was implemented to account for potential directional pleiotropy, using the inverse variance of the SNP–outcome association estimates as weights.^[19]^ Weighted median estimation provided an additional robustness check, producing valid estimates even when up to 50% of the IVs are invalid. The combination of these methods improves the robustness of the results and provides a more comprehensive assessment of causality.

### 2.6. Heterogeneity, pleiotropy, and sensitivity analysis

Heterogeneity among IVs was assessed using Cochran *Q* statistic, with *P* < .05 indicating significant heterogeneity and *P* > .05 suggesting no evidence of heterogeneity. The horizontal pleiotropic test was conducted using 2 methods: the intercept term test of MR-Egger regression. A intercept term *P *> .05 indicates no horizontal pleiotropic, and the result is reliable; for the global test of MR-PRESSO, a *P*-value < .05 indicates the presence of pleiotropic outliers, and the influence needs to be excluded through correction analysis. Sensitivity analysis employs the “leave-one-out” test. The principle is to gradually eliminate individual SNPs and then re-conduct IVW analysis to observe whether the causal effect values of the remaining SNPS show significant fluctuations, determine if there are any outliers that have a significant impact on the results, and verify the stability of the results.^[20]^

## 3. Results

**Figure 2.**
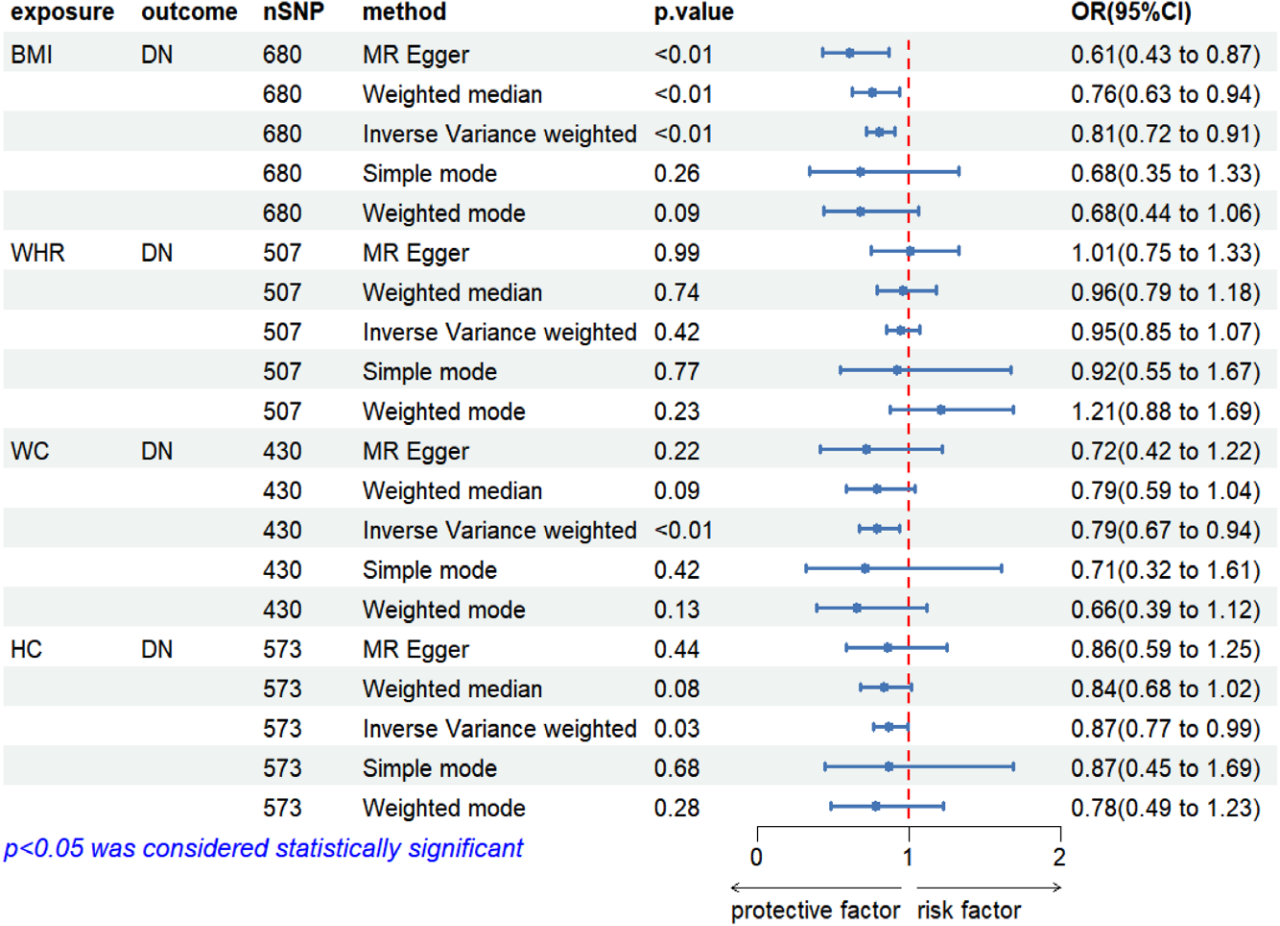
Forest plot of MR between BMI, WHR, WC, HC, and DN. BMI = body mass index, DN = diabetic neuropathy, HC = hip circumference, MR = Mendelian randomization, WC = waist circumference, WHR = waist-to-hip ratio.

MR analyzes were conducted using the TwoSampleMR package in R, with DN as the outcome variable and BMI, WHR, WC, and HC as the exposure variables. The primary causal effect estimates are summarized in a forest plot (Fig. 2).

### 3.1. SNP information for IVs

The exposure variables in this study (BMI, WHR, WC, and HC) were each analyzed separately with DN as the outcome in a 2-sample MR framework. Based on the predefined selection criteria, we identified 680 SNPs for BMI, 507 SNPs for WHR, 430 SNPs for WC, and 573 SNPs for HC as IVs. The average *F*-statistic corresponding to each exposure factor was all >10. The *R*^2^ values are 0.0017, 0.001, 0.0015, and 0.0069 respectively, suggesting that the IV is a “strong IV” and there is no bias towards weak IVs (Fig. 2).

### 3.2. Impact of BMI on DN

**Figure 3.**
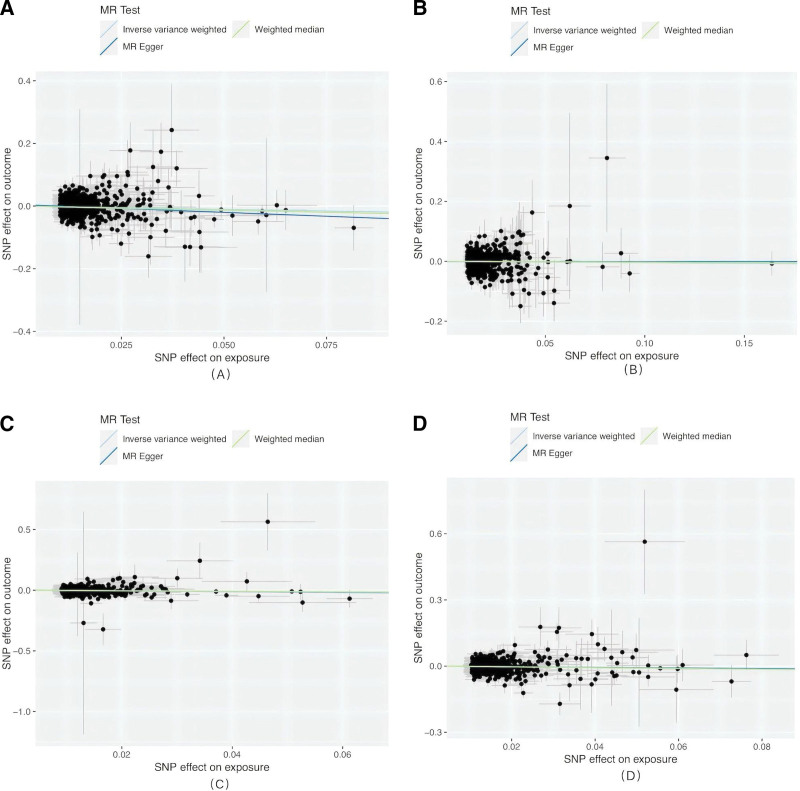
Scatterplots of MR analyzes (A–D: DN patients analyzed for BMI, WHR, WC, and HC). BMI = body mass index, DN = diabetic neuropathy, HC = hip circumference, MR = Mendelian randomization, WC = waist circumference, WHR = waist-to-hip ratio.

A total of 680 independent genome-wide significant SNPs associated with BMI were obtained from the GWAS dataset. All selected SNPs met the threshold for strong IVs (*F* = 44.75 > 10). Using the IVW method, BMI was found to have a significant protective causal effect against DN [OR = 0.808, 95% CI: 0.716–0.913, *P* = .0006]. After Bonferroni correction, it still had statistical significance (*P* = .003 < 0.0125) (Table 2, Fig. 3A).

**Table 2:** Effect of obesity on constipation risk (European population).

Exposures	Outcome	nSNP	Method	*P*-value	OR	OR_lci95	OR_uci95	*P*_Bonferroni
BMI	DN	680	MR-Egger	<.01	0.61	0.43	0.87	.035
		680	Weighted median	<.01	0.76	0.63	0.94	.041
		680	Inverse-variance weighted	<.01	0.81	0.72	0.91	.003
		680	Simple mode	.26	0.68	0.35	1.33	1.000
		680	Weighted mode	.09	0.68	0.44	1.06	.311
WHR	DN	507	MR-Egger	.99	1.01	0.75	1.33	.998
		507	Weighted median	.74	0.96	0.79	1.18	.987
		507	Inverse-variance weighted	.42	0.95	0.85	1.07	.987
		507	Simple mode	.77	0.92	0.55	1.67	.987
		507	Weighted mode	.23	1.21	0.88	1.69	.987
WC	DN	430	MR-Egger	.22	0.72	0.42	1.22	1.000
		430	Weighted median	.09	0.79	0.59	1.04	.488
		430	Inverse-variance weighted	<.01	0.79	0.67	0.94	.045
		430	Simple mode	.42	0.71	0.32	1.61	1.000
		430	Weighted mode	.13	0.66	0.39	1.12	.751
HC	DN	573	MR-Egger	.44	0.86	0.59	1.25	1.000
		573	Weighted median	.08	0.84	0.68	1.02	.398
		573	Inverse-variance weighted	.03	0.87	0.77	0.99	1.597
		573	Simple mode	.68	0.87	0.45	1.69	1.000
		573	Weighted mode	.28	0.78	0.49	1.23	1.000

BMI = body mass index, HC = hip circumference, MR = Mendelian randomization, SNP = single nucleotide polymorphism, WC = circumference, WHR = waist-to-hip ratio.

### 3.3. Impact of WHR on DN

A total of 507 independent genome-wide significant SNPs associated with WHR were identified from the GWAS dataset. All selected SNPs met the threshold for strong IVs (*F* = 62.91 > 10). Using the IVW method, no evidence of a causal relationship was observed between WHR and DN [OR = 0.954, 95% CI: 0.849–1.071, *P* = .425]. After Bonferroni correction, there was still no statistical significance (*P* = 1.000 > .0125) (Table 2, Fig. 3B).

### 3.4. Impact of WC on DN

We identified 430 independent genome-wide significant SNPs associated with WC from the GWAS dataset. These SNPs were used as IVs in the MR analysis, all classified as strong IVs (*F* = 44.53 > 10). IVW analysis indicated that WC had a significant protective causal association with DN [OR = 0.793, 95% CI: 0.666–0.943, *P* = .008]. After Bonferroni correction, there was no statistical significance (*P* = .046 > .0125) (Table 2, Fig. 3C).

### 3.5. Impact of HC on DN

A total of 573 independent genome-wide significant SNPs associated with HC were obtained from the GWAS dataset. All SNPs were considered strong IVs (*F* = 47.38 > 10). IVW analysis suggested a significant protective causal relationship between HC and DN [OR = 0.871, 95% CI: 0.768–0.988, *P* = .032]. After Bonferroni correction, there was no statistical significance (*P* = .159 > .0125) (Table 2, Fig. 3D).

### 3.6. Heterogeneity, pleiotropy, and sensitivity analysis

**Figure 4.**
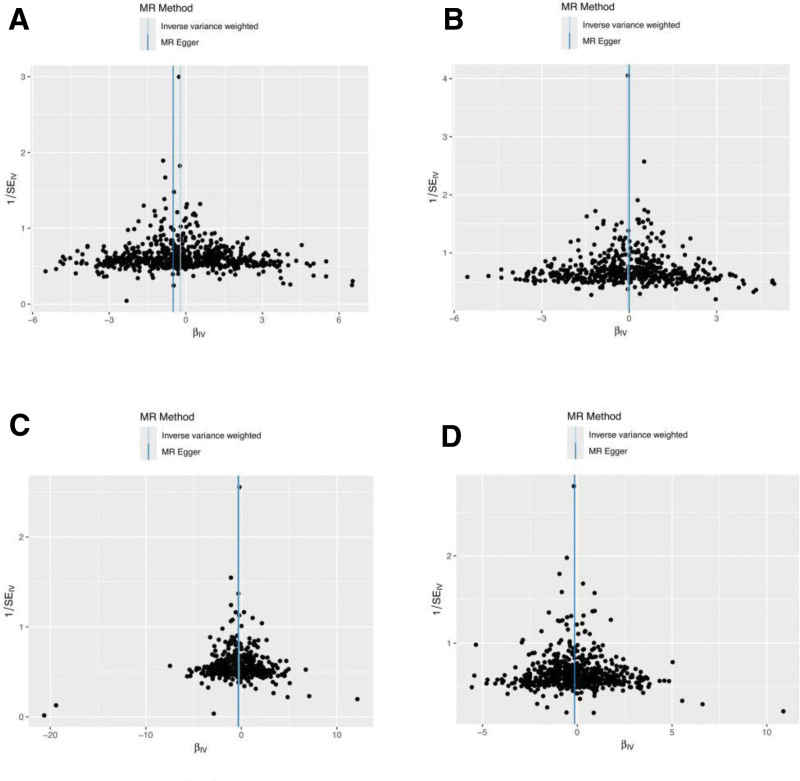
Funnel plots of MR analysis of heterogeneity test results (A–D: DN patients analyzed for BMI, WHR, WC, and HC). BMI = body mass index, DN = diabetic neuropathy, HC = hip circumference, MR = Mendelian randomization, WC = waist circumference, WHR = waist-to-hip ratio.

The likelihood of false-negative results was considered minimal, as this study strictly adhered to the IV selection criteria and included only participants of European ancestry. Heterogeneity was evaluated using Cochran *Q* test, which revealed that some MR analyzes exhibited significant heterogeneity (*P* < .05) (Table 3, Fig. 4). Although the datasets were derived from large, well-established consortia such as the GWAS Catalog and FinnGen, potential sources of bias remain (stemming from differences in genotyping platforms, population characteristics, and phenotypic definitions) which may have contributed to heterogeneity in the analyzes.

**Table 3:** Effect of obesity on constipation risk (European population).

Exposures	Outcome	No. of SNPs	Modes	OR (95% CI)	*P*-vaule	MR-Egger pleiotropy test		Global test
						Intercept	*P*-value	*P*-value
BMI	DN	680	MR Egger	0.61 (0.43–0.87)	<.01	0.005	.1	.003
WHR	DN	507	MR Egger	1.01 (0.75–1.33)	.99	<0.001	.967	.42
WC	DN	430	MR Egger	0.72 (0.42–1.22)	.22	0.001	.699	.002
HC	DN	573	MR Egger	0.86 (0.59–1.25)	.44	<0.001	.718	.005

The intrefer to MR-Egger intercept. *P* pleiotropy refer to MR-Egger intercept’s *P*-value.

BMI = body mass index, DN = diabetic neuropathy, HC = hip circumference, MR = Mendelian randomization, SNP = single nucleotide polymorphism, WC = circumference, WHR = waist-to-hip ratio.

Horizontal pleiotropy: MR-Egger intercept shows, across all exposures (BMI, WHR, WC, and HC), the Egger intercept values were close to 0, and the corresponding *P*-values exceeded .05, indicating no evidence of horizontal pleiotropy in any of the MR analyzes; the global test *P *< .05 of the MR-PRESSO test indicates the presence of non-directional pleiotropy. However, the outlier test did not identify specific significant outlier SNPS, suggesting that the pleiotropy stems from randomly distributed minor anomalies rather than a single strongly influential SNP. To verify the stability of the results, we further conducted a sensitivity analysis using the leave-one-out method (Table 3).

**Figure 5.**
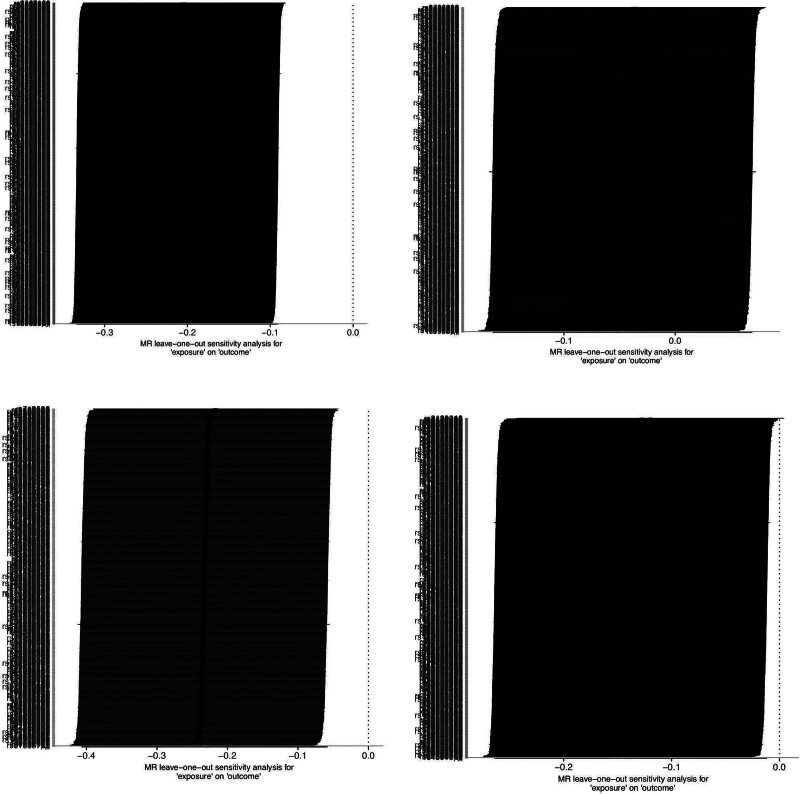
MR leave-one-out sensitivity analysis. MR = Mendelian randomization.

Sensitivity analyzes were performed using the leave-one-out method. Sequential removal of individual SNPs showed that the IVW effect estimates of the remaining SNPs exhibited no substantial fluctuations and remained closely aligned with the positions of the red dots in the corresponding plot. These findings indicate that no single SNP among the selected IVs had a disproportionate influence on the causal estimates, thereby supporting the stability and robustness of the IVW results (Fig. 5).

## 4. Discussion

In this study, we applied MR using large-scale GWAS summary statistics to explore the causal relationship between obesity and DN. Due to the high correlation of the 4 exposure relationship traits, there is potential multicollinearity. We used univariate MR to analyze the independent effects of each trait respectively, and the bias of multiple tests was controlled by Bonferroni correction. It can still provide reliable evidence for the causal relationship between obesity and DN. When controlling the LD degree among SNPs, if the threshold is too high (*r*^2^ > 0.1), some associated SNPs may be retained, resulting in non-independence among IVs and increasing the risk of bias in the analysis results. If the threshold is too low (*r*^2^ < 0.001), although it can ensure the independence of SNPs, it may screen out a large number of useful SNPs, resulting in too few available IVs in the end and affecting the statistical power. Meanwhile, the number of SNPs we screened out was not large. To ensure there were sufficient IVs for subsequent analysis, the researchers would appropriately relax this threshold. Therefore, in this study, the threshold *r*^2^ < 0.01 was adopted. Finally, our findings revealed a significant negative causal association between genetically predicted BMI and the risk of DN, with no significant relationship for WHR, WC, and HC. These results provide genetic evidence that certain obesity-related traits may exert a protective effect against DN, offering new perspectives for prevention and treatment strategies.

Previous observational studies have reported inconsistent associations between obesity and DN: some identifying positive correlations,^[21–23]^ others negative.^[1,24–26]^ By leveraging genetic instruments, MR minimizes confounding and reverse causation, strengthening causal inference. A prospective cohort study found that BMI was positively associated with DN before diabetes onset but inversely associated afterward, suggesting weight management may be beneficial in diagnosed patients.^[1]^ Valensi et al^[26]^ similarly reported a negative association between BMI and cardiac autonomic neuropathy. These findings align with the “obesity paradox” in type 2 DM, where overweight or obese individuals have lower mortality risk than those of normal weight.^[27,28]^ Possible explanations include protective adipokines that reduce neuroinflammation, earlier diagnosis and treatment among obese individuals, and metabolic adaptations that may slow DN progression. However, this apparent protection could partly reflect earlier intervention rather than obesity itself,^[29]^ and some obesity-related genetic variants may influence DN via alternative pathways independent of adiposity.

The mechanisms linking obesity and DN are complex. Adipose tissue acts as an endocrine organ, secreting bioactive adipokines that can improve insulin sensitivity and reduce inflammation. Obesity-related nerve injury may also be influenced by the structural characteristics of the peripheral nervous system, which contains long axons requiring continuous support from neuroglia. Emerging evidence suggests that extracellular vesicles released by neuroglia can carry bioactive molecules that help maintain neuronal function and integrity.^[30]^ In DN and peripheral nerve injury, growth factor expression is reduced, limiting neuronal repair and regeneration.^[31]^ In addition, obesity-related nerve injury involves oxidative stress, chronic inflammation, and microvascular dysfunction.^[32,33]^ Activated macrophages release cytokines and chemokines that exacerbate oxidative stress and nerve fiber damage.^[33,34]^

Obesity is also a major component of metabolic syndrome, a recognized risk factor for peripheral and autonomic neuropathy.^[35–38]^ Studies indicate obesity may increase parasympathetic dysfunction risk, particularly in men, and contribute to early cardiac autonomic neuropathy pathogenesis.^[39–41]^ The discrepancy between our MR results and much of the observational literature may arise from the inability of traditional studies to fully control for confounders. MR analysis, by using genetic variants as IVs, reduces bias and reverse causation. Nonetheless, the relationship between obesity and DN is likely multifactorial and stage-dependent, consistent with the obesity paradox observed in other chronic conditions.

## 5. Limitations and prospects

Limitations of this study include potential pleiotropy, which could allow genetic variants to influence DN through non-obesity pathways, and restriction to European ancestry populations, limiting generalizability. Future research integrating multi-omics approaches (such as transcriptomics and proteomics) could clarify molecular mechanisms and pathways linking obesity to DN. Larger, multiethnic MR analyzes and prospective cohort studies that account for environmental and lifestyle factors are warranted to validate and extend these findings.

## 6. Conclusions

This MR study provides genetic evidence supporting a causal and inverse association between body mass index, and the risk of DN, while no causal relationship was observed for WHR, WC, and HC. These findings highlight the complex interplay between obesity-related anthropometric traits and DN and underscore the need for further mechanistic research to elucidate the underlying biological pathways.

## Acknowledgments

We appreciate the data access to The NHGRI-EBI Catalog of human genome-wide association studies (GWAS Catalog) and FinnGen database.

## Author contributions

**Conceptualization:** Zhuolin Wang.

**Data curation:** Cong Li, Lihui Wang.

**Methodology:** Chunmei Dai.

**Validation:** Mingquan Li.

**Writing – original draft:** Xiaoting Zhu, Chengyan Wang.

**Writing – review & editing:** Chunmei Dai, Mingquan Li.
